# Visual snow syndrome: A clinical and phenotypical description of 1,100 cases

**DOI:** 10.1212/WNL.0000000000008909

**Published:** 2020-02-11

**Authors:** Francesca Puledda, Christoph Schankin, Peter J. Goadsby

**Affiliations:** From the Headache Group (F.P., P.J.G.), Department of Basic and Clinical Neuroscience, Institute of Psychiatry, Psychology & Neuroscience, King's College London; NIHR-Wellcome Trust King's Clinical Research Facility (F.P., P.J.G.), SLaM Biomedical Research Centre, King's College Hospital, London, UK; and Department of Neurology (C.S.), Inselspital, Bern University Hospital, University of Bern, Switzerland.

## Abstract

**Objective:**

To validate the current criteria of visual snow and to describe its common phenotype using a substantial clinical database.

**Methods:**

We performed a web-based survey of patients with self-assessed visual snow (n = 1,104), with either the complete visual snow syndrome (n = 1,061) or visual snow without the syndrome (n = 43). We also describe a population of patients (n = 70) with possible hallucinogen persisting perception disorder who presented clinically with visual snow syndrome.

**Results:**

The visual snow population had an average age of 29 years and had no sex prevalence. The disorder usually started in early life, and ≈40% of patients had symptoms for as long as they could remember. The most commonly experienced static was black and white. Floaters, afterimages, and photophobia were the most reported additional visual symptoms. A latent class analysis showed that visual snow does not present with specific clinical endophenotypes. Severity can be classified by the amount of visual symptoms experienced. Migraine and tinnitus had a very high prevalence and were independently associated with a more severe presentation of the syndrome.

**Conclusions:**

Clinical characteristics of visual snow did not differ from the previous cohort in the literature, supporting validity of the current criteria. Visual snow likely represents a clinical continuum, with different degrees of severity. On the severe end of the spectrum, it is more likely to present with its common comorbid conditions, migraine and tinnitus. Visual snow does not depend on the effect of psychotropic substances on the brain.

Visual snow (VS) is a recently identified neurologic condition consisting of a constant positive visual disturbance described as uncountable tiny dots over the entire visual field^1^ (figure 1). In addition to the static, patients very often report visual symptoms such as palinopsia, entoptic phenomena, photophobia, and nyctalopia. This constitutes the VS syndrome (VSS), which is outlined by a set of specific criteria (table 1).^2^ From the first case report of VS by Liu et al.^3^ in 1995, the recognition of the disorder has grown considerably, to the point where VS is now included in the appendix of the International Classification of Headache Disorders as a complication of migraine.^4^

**Figure 1:**
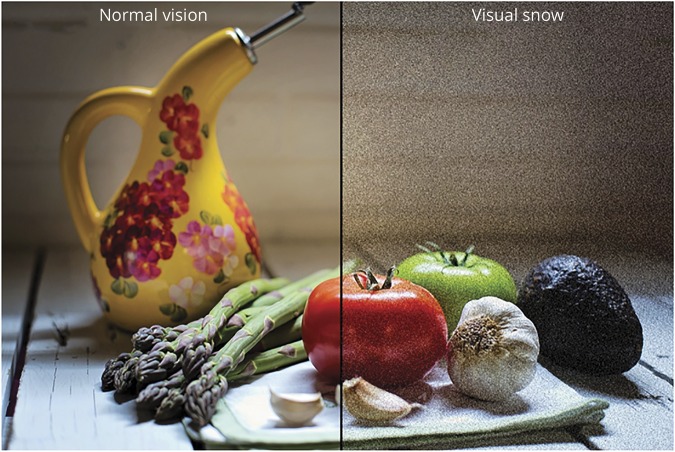
Illustration of visual snow

**Table 1:**
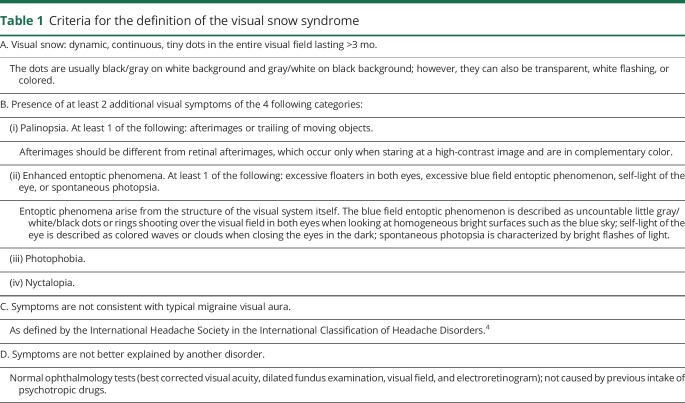
Criteria for the definition of the visual snow syndrome

Several questions concerning VS have arisen as the condition has become better known. For example, if there is a need to define the differences between VS and the presentation as a “complete” syndrome? What are its most common clinical phenotypes, and what is the relationship with other comorbid conditions? Similarly, given that hallucinogenics can produce a similar disturbance,^5^ are there clinical distinctions that may inform understanding the biology involved?

Here, we describe the clinical characteristics of a substantial population of patients with VS, both with and without the complete VSS. Our main objective was to test the current criteria and to confirm the typical presentation of the main symptom of VS—i.e., the static—in the context of a larger cohort and to begin to determine any broad differences related to geography. We also wanted to explore a dataset large enough to dissect possible subgroups and endophenotypes. Finally, we wished to observe the interaction between VS and its main comorbid conditions migraine and tinnitus and to compare the disturbance with patients with hallucinogen persisting perception disorder (HPPD).

## Methods

### Participant selection and recruitment

The study was advertised on the website of Eye On Vision (eyeonvision.org/), a patient self-help group for VS with whom we have collaborated. Most of the patients involved in the study approached our group through a dedicated research e-mail, which they could find on the website. A smaller number of patients had contacted the researchers individually asking to be involved in research and were redirected to the website.

An online survey was prepared in collaboration with the patient group and was made available on the Eye On Vision website. The survey is illustrated in table 2; it presents a series of open and dichotomous questions aimed to characterize the symptoms of VS following the available criteria. We also investigated the presence of migraine and tinnitus. Finally, we enquired about age at symptom onset and previous exposure to recreational drugs.

**Table 2:**
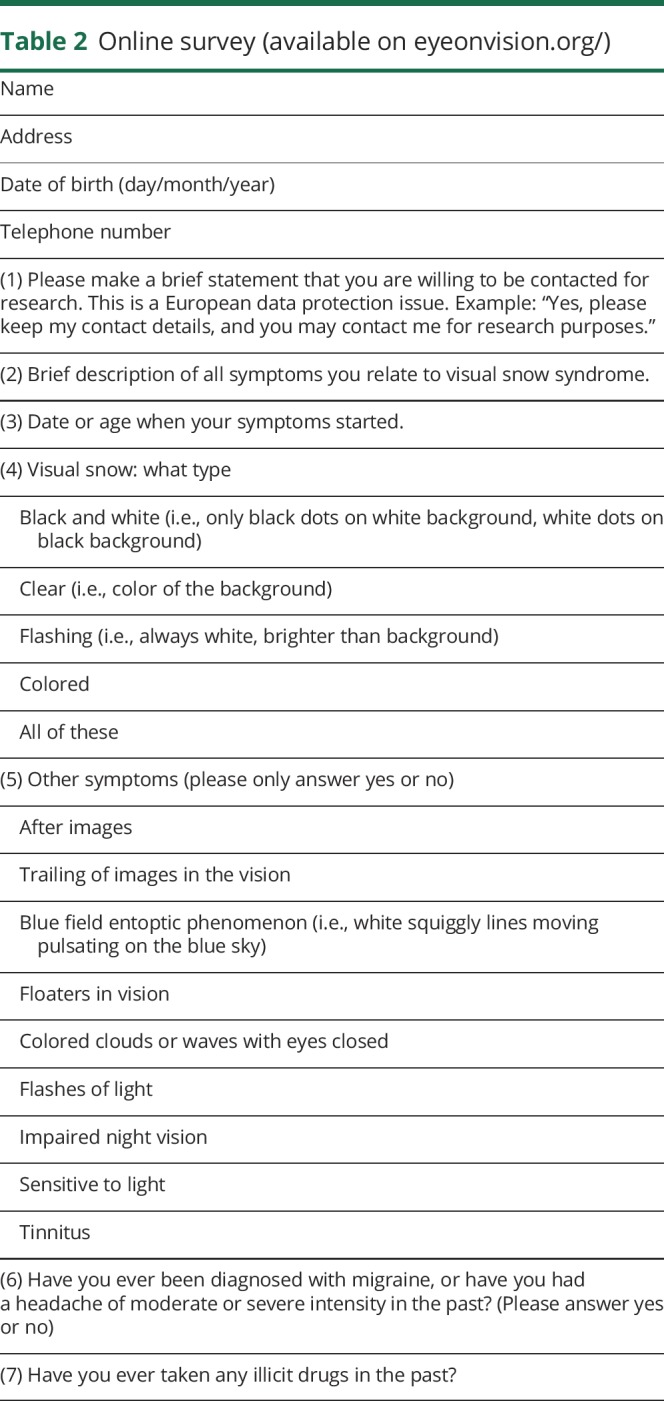
Online survey (available on eyeonvision.org/)

The study was approved by the KCL Research Ethics Panel. Data were collected between April 2016 and May 2018.

### Patient characterization

Following the 2014 criteria (table 1), we defined patients who self-reported visual symptoms corresponding to criterion A (as evaluated by responses to questions 2–4 of our survey) and who also fit criterion B (as evaluated by responses to question 5 in our survey) as having VSS. Criteria C and D were evaluated on a case-to-case basis on the basis of answers to questions 2, 6, and 7 and eventual follow-up questions by the investigators when in doubt. Patients who did not report >2 additional visual symptoms of the 4 main categories, therefore lacking criterion B, but who fit all the other criteria were considered to have VS without the syndrome.

To avoid confounding with HPPD, patients who answered “yes” to question 7 in our survey were further followed up with in-depth questions aimed at assessing when their symptoms appeared with respect to the intake of recreational drugs. All participants who reported the onset of VS symptoms in the 12 months after any exposure to recreational drugs were excluded from the first 2 groups, regardless of the remaining symptoms, and were added to a third group called possible HPPD. All participants in this third group fit criterion A for typical VS and were therefore included in the analysis.

All data collection and patient characterization were performed by one of us (F.P.).

### Statistical analysis

Data were tabulated (Excel 2016 for Windows). Descriptive statistics, analysis of variance, or χ^2^ analysis for comparisons of continuous and categorical variables and cluster analysis were performed with SPSS Statistics Version 24.0 for Windows (IBM Corp, Armonk, NY). Regression analysis, multiple imputations, and latent class analysis were performed in Stata (Stata Statistical Software release 15, 2017, StataCorp LLC, College Station, TX). Values of *p* < 0.05 were considered significant.

We separated patients into 3 different groups according to their diagnosis: patients with VSS were coded as group 1, patients with VS as group 2, and patients with HPPD as group 3.

An ordinal variable was created to measure disease severity according to the number of visual symptoms experienced. For the largest cohort (i.e., group 1), this outcome variable was regressed on selected variables using ordinal logistic regression. The variables included as covariates in this model were selected in a previous step based on a correlation with the number of symptoms experienced defined by significant correlations at the 5% level using the Spearman correlation.

A latent class analysis was performed to investigate possible endophenotypes of VS, first on group 1 only and then on groups 1 and 2 combined.

### Data availability

The data that support the findings of this study are available from the corresponding author on reasonable request.

## Results

### Demographic characteristics

From April 2016 to May 2018, patients (n = 1,400) contacted the study group through the e-mail designated to VS research. Of these, 210 either gave incomplete data in the initial survey or never replied after having been redirected to the patient website; these individuals were excluded from further data collection. Two individuals had an insufficient English level; 6 had a serious underlying ophthalmic condition; and 8 did not fulfill criterion A, meaning the static they reported either was of episodic nature or was present in only 1 part of the visual field. These patients were all excluded from the analysis.

Demographic characteristics of the remaining participants (n = 1,174) are presented in table 3, which also shows details of symptom onset and associated comorbid conditions. The majority of the cohort (n = 1,061, 90%) had complete VSS. Forty-three participants in the cohort were considered to have VS alone because they provided a very clear description of the dynamic continuous pan-field tiny dots described in criterion A. These participants, however, did not present at least 2 visual symptoms from the additional categories and were grouped in the VS category. Of these, 7 participants had no additional symptoms, 20 had only 1 symptom, and 16 had between 2 and 4 symptoms of the same category, e.g., palinopsia for stationary objects and trailing or several entoptic phenomena.

**Table 3:**
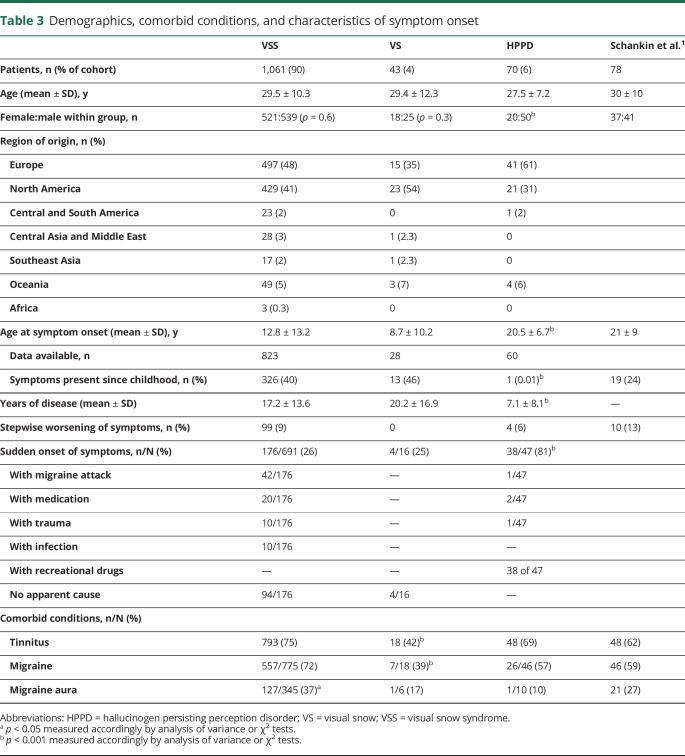
Demographics, comorbid conditions, and characteristics of symptom onset

Seventy participants were grouped as possible HPPD following the criteria previously described.^1^ With the exception of exposure to recreational drugs in the 12 months before the onset of symptoms, they all had the remaining criteria for VSS diagnosis.

### Features of the disorder in the cohorts and comparison to HPPD

In table 3, the 3 groups are compared among themselves and with the cohort from the 2014 study, when the required data were available. The 4 groups did not differ with regard to age. Male and female ratios were similar for participants with VS (VSS and VS); however, in the HPPD population, most patients (71%; *p* < 0.001) were male. The large majority of participants came from North America (n = 429, 41% for VSS; n = 23, 54% for VS; n = 21, 31% for HPPD) and Europe (n = 497, 48% for VSS; n = 15, 35% for VS; n = 41, 61% for HPPD).

Participants with HPPD had a significantly later onset of symptoms compared to patients with VS, both with and without the syndrome (*p* < 0.001). As a consequence, the average years with disease were lower in the HPPD group (*p* < 0.001). A small number (n = 99) of participants in the VSS group reported a clear stepwise worsening of symptoms at some point of the condition; however, this feature was not routinely screened for, so it is not possible to infer its actual prevalence. Forty percent of patients with VSS for whom data on onset age were available reported the presence of symptoms since childhood, meaning for as long as they could recall. This was higher than the proportion found in the 2014 study. About one-quarter of the participants with VS (VSS and VS) reported a sudden onset of their symptoms; however, the real frequency of this form of onset might be different because participants were not interrogated about it directly. Of these spontaneous reports of sudden onset, some were related to specific conditions indicated in the table 3; a migraine attack was the most frequent. In the majority of cases, however, the participants could not recall any specific associated event. A sudden onset of symptoms was significantly more frequent in the HPPD group (81%; *p* < 0.001). By definition, all of these patients had the start of symptoms within a year after using recreational drugs. Four of these participants could also recall other specific events (i.e., a migraine attack, a new medication, and a mild head trauma) in strict temporal relation to the beginning of their symptoms.

### Tinnitus and migraine

The presence of tinnitus in the VSS population was the highest but overall similar to that of the HPPD and 2014 cohorts. The frequency of this symptom was, however, significantly lower in the VS group compared to the others. This was also the case for migraine, which was significantly less frequent in the VS population and more frequent in the VSS population. The presence of migraine aura was not routinely investigated because we considered this diagnosis unreliable for a dichotomous questionnaire. It was spontaneously reported in 37% (*p* = 0.05) of participants in the VSS group, and in all these cases, the diagnosis was confirmed with thorough follow-up questions.

### Clinical characterization of VS: Static

We collected information on the type of static that patients experienced and the associated visual symptoms that form the VSS. Table 4 shows the frequencies of these characteristics, comparing them across the 3 groups and with the 2014 study cohort.

**Table 4:**
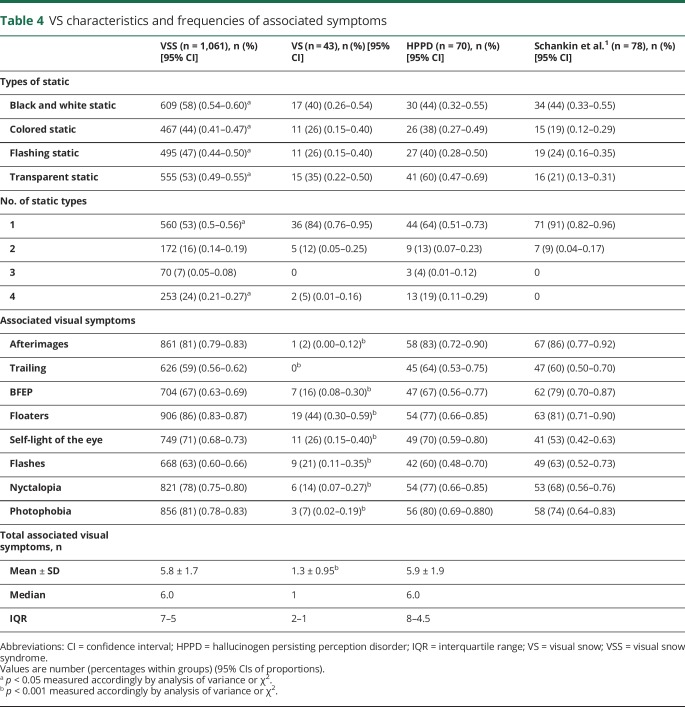
VS characteristics and frequencies of associated symptoms

Each of the 4 static types was reported more frequently in the VSS group. This is probably accounted for by the fact that this group had a significantly higher proportion of participants reporting all 4 types of static. The most common type of static differed between the VSS and VS groups compared to the HPPD group. When only 1 type of static was present, this was most commonly black and white in the VSS and VS groups and transparent in the HPPD group. When 2 types of static were present, the most frequent combination was black and white and transparent for the VSS and VS groups and colored and flashing for the HPPD group. When 3 types of static were present, the combination of black and white, flashing, and transparent was the most common in the VSS and VS groups, whereas the combination of colored flashing and transparent was the most common in the HPPD group. The 2014 cohort showed different results from all groups in the present study, with no patients reporting a combination of >2 types of static. This could be due to the fact that the current questionnaire was more flexible on static types, accounting for all types as defined by criterion A.

### Clinical characterization of VS-associated visual symptoms

With regard to the associated visual symptoms, the VSS and HPPD groups did not significantly differ with regard to the mean number of associated visual symptoms reported by each patient. The 3 most common symptoms in the VSS group were, in order, floaters, afterimages, and photophobia. In the VS cohort, the most frequent symptoms were all of the entoptic phenomena group: floaters, self-light of the eye, and flashes. In the HPPD group, the 4 most commonly reported symptoms were afterimages, photophobia, floaters, and nyctalopia. Taking each symptom separately, the VSS and HPPD groups did not differ among themselves or with the 2014 cohort with regard to the frequencies of each symptom.

### Predicting severity based on symptoms: Ordinal logistic regression

The results are shown in table 5. We first performed a complete case analysis in which any case with a missing value on either the outcome or the covariates was omitted from the analysis. We then performed the same analysis using 10 imputed datasets in which a chained equations approach was used and the imputations were stratified by sex. The parameter estimates were similar across both models. This is unsurprising because there were no missing data for the symptoms and the level of missing data was low for most covariates (<2%) except for disease duration and presence of migraine. These 2 variables were added later to the data collection procedure and thus likely to be missing at random. One participant for whom sex was not specified was excluded from the analysis.

**Table 5:**
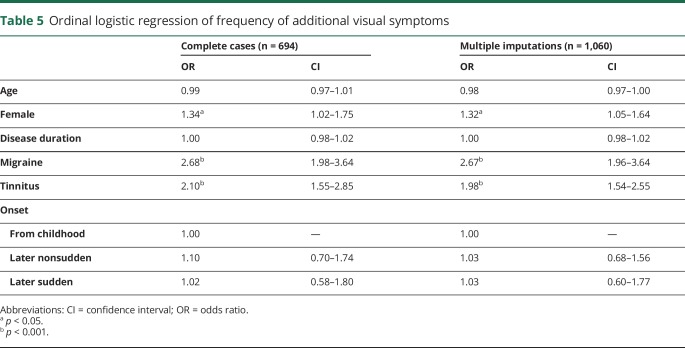
Ordinal logistic regression of frequency of additional visual symptoms

The results from the multiply imputed analysis indicated that female participants were approximately one-third more likely to experience a higher number of symptoms than male participants. Those reporting migraine were >2.5 times more likely to experience a higher number of symptoms, and those with tinnitus were about twice as likely to experience a higher number of symptoms. A test for an interaction between migraine and tinnitus was not significant, indicating that these 2 concomitant conditions exert independent and additive effects on the number of VS symptoms experienced. Age, disease duration, and type of onset were not related to the number of symptoms experienced.

### Latent class analysis

**Figure 2:**
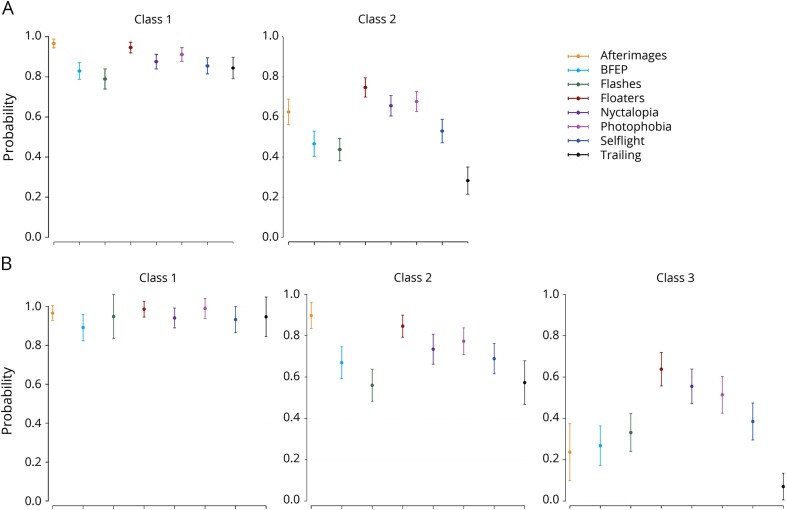
Latent class analysis (A) Latent class analysis performed on n = 1,060 patients with complete visual snow (VS) syndrome (VSS). Model fit criteria (table 6a) suggested that a 2-class solution best explained the data. The latent classes, which separated the patients into groups based on additional visual symptom frequency, are shown below. (B) Latent class analysis performed on n = 1,104 patients with complete VSS and VS without the syndrome. Patients with hallucinogen persisting perception disorder were excluded. With the addition of patients with VS, an extra class was recovered (classes 1–3 shown below). However, the model still separated the patients into groups based on additional visual symptom frequency only. BFEP = blue field entoptic phenomenon.

The results of the latent class analysis are shown in table 6 and figure 2, A and B. This was performed first only on participants with VSS (table 6a), with the exclusion of 1 participant for whom sex was not defined (n = 1,060). Model fit criteria suggested that a 2-class solution provided the most parsimonious explanation of the data, where classes 1 and 2 accounted for x and y of the sample, respectively. The classes that were obtained separated the patients into groups based on additional visual symptom frequency. Logistic regression indicated that the same variables as the ordinal logistic regression for symptom frequency were related to latent class membership (table 6b).

**Table 6:**
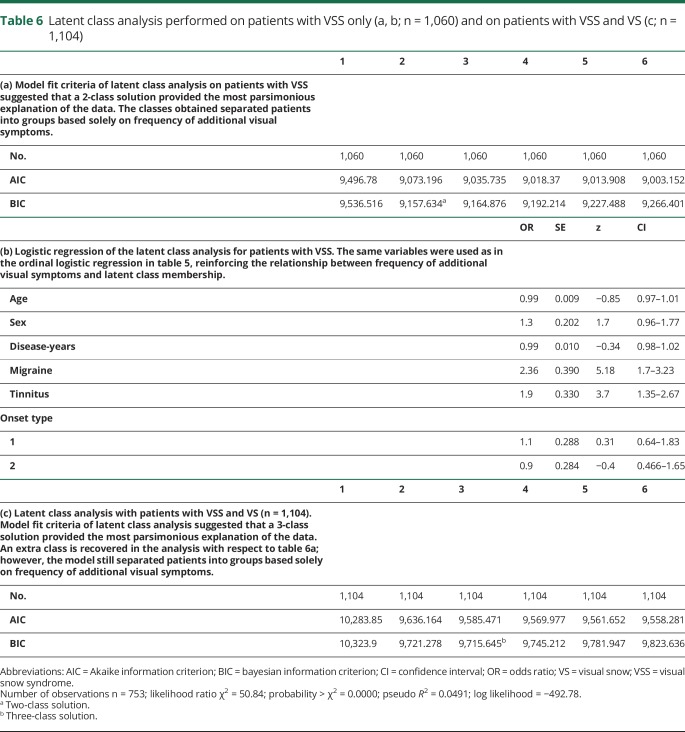
Latent class analysis performed on patients with VSS only (a, b; n = 1,060) and on patients with VSS and VS (c; n = 1,104)

We then performed the same analysis on participants with VSS and VS (n = 1,104; table 6c), excluding only those with HPPD. The analysis showed that a third latent class was obtained with this further step; however, the model still showed a group separation based solely on additional visual symptom frequency.

## Discussion

We here describe and analyze a large cohort of patients with VS. The data support VSS as a well-delineated, clinically recognizable disorder. Most patients have VS with other visual symptoms. There is an association with migraine and tinnitus that is independent, and the condition is clearly not simply due to hallucinogenic intake. The size of our patient sample and its provenance from different parts of the world make it representative of the real population and allow some inference on several aspects of this condition that should facilitate and guide further study of the problem.

The results from this study indicate that participants with VSS are usually young and most commonly present with black and white or transparent static, as well as a high number of additional visual symptoms. Even if there is no specific sex prevalence, identifying with female sex is significantly associated with reporting increased severity of the condition. The visual static can occur in different combinations of color. Visual disturbances can also present in several combinations. However, floaters, afterimages, and photophobia are almost invariably present and might in fact constitute a hallmark of the syndrome. The disorder usually starts in early life, and in many cases, the patients have it since childhood and can never recall seeing differently. In these cases, the affected person can find out almost serendipitously about the anomaly in seeing VS, usually by comparison with unaffected family members or friends. In a significant number of patients, VS can start abruptly and spontaneously; however, this is not necessarily related to a higher number of symptoms in the condition.

This study has shown that once specific criteria are defined and followed,^1^ VS is a recognizable disorder, with a very homogeneous clinical presentation. The description of the primary symptom (i.e., the static) was highly reproducible across our cohort, with only a few participants actually presenting a visual disturbance not attributable to VS. The overall clinical presentation was also quite similar across our participants (see previous paragraph) and with the second largest cohort in the literature,^1^ albeit with some variations perhaps attributable to different sample sizes or different methodology. For the 2014 study, patients were in fact interviewed in detail via telephone, whereas the present study was questionnaire based.

The current criteria for the syndrome usefully eliminate false-positive participants. In fact, only a small minority of self-reporting patients consecutively recruited in the present study did not fit the full syndrome definition. It is nonetheless important to recognize the presence of VS in these patients even in the absence of additional visual disturbances, which both characterize a higher severity and define the syndrome but are not a sine qua non for VS itself. In fact, VS is likely to constitute a spectrum type of disorder, with patients ranging in severity. In this context, a severe end of the spectrum could be represented by those patients who have the static with all the visual disturbances and are highly affected by them, and a mild end could be represented by those who have only static and are not bothered by it, possibly even considering it normal for most of their lives. This theory is reinforced by the fact that VSS and VS did not differ in their key clinical features such as average age, sex distribution, mode, and age at onset. They did, however, differ when it came to comorbid conditions, which were more likely to be found within the syndrome in patients with a higher number of associated symptoms (i.e., a more severe condition) and were less frequent in patients with VS but no syndrome compared to patients with VSS.

This is emphasized by the latent class analysis itself showing that VS does not present with specific clinical endophenotypes and is classified predominantly on its severity (measured with the burden of additional symptoms). Further studies with objective measures on the levels of static such as severity and disability scales as perceived by patients would be needed to confirm this theory.

VS has 2 main comorbid conditions, migraine and tinnitus.^6–12^ This strongly reported association suggests that these 2 conditions might share some common pathophysiologic mechanism with VS. This hypothesis is substantiated by a study that investigated brain metabolism in 17 patients with VS with the use of [^18^F]-fluorodeoxyglucose-PET^6^ and showed a hypermetabolism of the right lingual gyrus in patients with VS. The area of the lingual gyrus corresponds to Brodmann area 19 in the supplementary visual cortex and is pivotal in processing complex downstream visual inputs. This area is also involved in photophobia in migraine,^13^ which further corroborates the concept of a shared pathophysiology between migraine and VS, possibly on the basis of a dysfunctional cortical mechanism common to both conditions.

Here, we confirmed the presence of these comorbid conditions in a larger sample of patients with VS and demonstrated that both conditions are associated with a worse presentation of VS, defined by having more additional visual symptoms. These comorbid conditions independently predicted the affinity to a severity class in the latent class model and the number of additional symptoms in the ordinal logistic regression. This confirms the clinical and pathophysiologic importance of interaction between migraine, tinnitus, and VS.

Tinnitus is a common disorder in the general population, with a prevalence ranging between 5% and 25%.^14–17^ In the present cohort, three-quarters of patients with VSS also had tinnitus, suggesting a more than chance association between the 2 conditions. On a theoretical basis, VS and tinnitus represent 2 different manifestations of a similar disorder, which is the perception of a sensory stimulus that is not present or is subthreshold. This neurobiological dysfunction would probably point to a central neuronal mechanism, which could involve aberrant sensory processing at the level of association cortices or the thalamo-cortical network.

Given that tinnitus not only is more frequently present but also predicts the severity of VS, it is possible that both disorders share a common pathophysiologic mechanism, which, if sufficiently active, can manifest clinically with both conditions. This is what seems to happen in the majority of the patients in the present study. This hypothesized mechanisms could involve thalamo-cortical dysrhythmia^18,19^ and cortical disexcitability, both of which have also been largely implicated in the context of migraine pathophysiology.^20,21^ A possible confirmation of such hypothesis comes from a very recent neurophysiologic study that showed late visual evoked potential alterations in VS highly suggestive of a visual association cortex dysfunction.^22^ Therefore, VS could represent an abnormality of sensory perception, potentially involving multiple senses simultaneously.

A dishabituation mechanism common to migraine and VS would explain the worsening of the VS condition when migraine is present, which was found here and in previous studies,^6^ as well as the strong comorbidity between them. The presence of associated visual symptoms, enhanced entoptic phenomena in particular, also potentially points to a disorder of habituation and sensory processing, which allows the perception of stimuli that are normally ignored by the brain. A migrainous pathophysiology alone, however, is not sufficient to explain the VS biology, primarily because of the chronic nature of this disorder as opposed to the ictal features of migraine, but also because most preventive migraine medication used empirically shows very little effect on VS.^23^

An important issue with VS is the assumption that it could be due to HPPD. HPPD is a condition codified in DSM-V^24^ and characterized by the re-experiencing of perceptual symptoms (flashbacks), typically of the visual type, that follow the cessation of the use of a hallucinogen and were experienced during the intoxication.^25^ VS and HPPD indeed share some clinical aspects, mostly characterized by the possibility of the latter to manifest with visual static, palinopsia, flashes, and other types of visual dysperceptions.^5,25,26^ Recent literature seems to suggest that HPPD can be distinguished into 2 main entities. In type 2 HPPD, the visual symptoms are constant or nearly constant,^27^ consistent with the group of participants from our cohort. In this study, to avoid any possible confounding overlap between HPPD and VS, we applied strict criteria to identify VSS and VS. We considered 12 months from the intake of any recreational drugs as an appropriate time to exhaust possible effects of psychotropic substances on the visual system. Any participants exposed within this time frame were excluded from the VS groups. We believe this allowed us to confirm that VS pathophysiology does not have a connection with the use of recreational substances. Furthermore, we were able to analyze a third group of patients presenting with the VSS phenotype but for whom HPPD could not be excluded. These participants were mostly male, which might be due to substance use being generally more common in men than women,^28^ and exhibited a later onset of VS symptoms, in most cases with an abrupt start. However, they fulfilled all remaining criteria for the syndrome and did not differ from the main VSS group with regard to clinical VS characteristics. Studies with confirmed HPPD would be necessary to shed more light on the interesting overlap between VSS and drug intake, which may indeed represent different aspects of a same disorder or 2 distinct conditions with shared pathophysiologic mechanism. Our data do suggest that VSS itself is not part of HPPD but rather that HPPD can manifest in the VSS clinical spectrum.

There are important limitations to this work largely centered around recruitment bias. First, recruited patients had contacted the group directly to be involved in research; this is likely to have selected participants at the more severe end of the clinical spectrum. Nonetheless, most participants were not seeking medical help when they contacted us, stating that their primary reason for contact was simple curiosity about their disorder. The fact that access to the study was solely through the internet might have also biased toward a younger population. The absence of an objective measure of clinical severity is another limitation to this study; such measures are yet to be developed. Finally, the methodology was based on questionnaires completed remotely and relied heavily on patient participation. The absence of a structured interview conducted by a physician might have hindered the clinical description in some cases. However, using a web-based survey allowed broader geography that simply is not feasible with in-person approaches. Moreover, the web-based approach guaranteed the largest possible participation in the study, which again would be very challenging if all cases were to have had telephone interviews. The combination of detailed phenotyping in person and hypothesis testing using broad, internet-based samples offers a powerful tool, particularly when studying a relatively poorly characterized condition.

VSS is a geographically widely distributed neurologic disorder that can be consistently defined by the current diagnostic criteria. VSS does not manifest with specific endophenotypes and likely represents a clinical continuum, with patients ranging in different degrees of severity. On the severe end of the spectrum, VS is more likely to present with its common comorbid conditions, migraine and tinnitus. VSS is independent of the use of hallucinogenic substances, although HPPD can manifest in the VS spectrum. In the future, further studies are needed to enhance our understanding of the underlying neurobiology of VS and consequently to move toward an era of better management of the condition.

## Glossary

DSM-V: *Diagnostic and Statistical Manual of Mental Disorders, 5th edition*
HPPD: hallucinogen persisting perception disorder
VS: visual snow
VSS: visual snow syndrome
